# Theory’s role in shaping behavioral health research for population health

**DOI:** 10.1186/s12966-015-0307-0

**Published:** 2015-11-26

**Authors:** Abby C. King

**Affiliations:** Division of Epidemiology, Department of Health Research & Policy, Stanford University School of Medicine, 259 Campus Drive, HRP Redwood building, Room T221, Stanford, CA 94305-5405 USA; The Stanford Prevention Research Center, Department of Medicine, Stanford University School of Medicine, 259 Campus Drive, HRP Redwood building, Room T221, Stanford, CA 94305-5405 USA

**Keywords:** Behavioral health research, Theory, Ecological, Systems science, Citizen science, Population health

## Abstract

The careful application of theory often is used in the behavioral health field to enhance our understanding of how the world currently works. But theory also can help us visualize what the world can become, particularly through its potential impacts on population-wide health. Applying a multi-level ecological perspective can help in expanding the field’s focus upward toward the population at large. While ecological frameworks have become increasingly popular, arguably such perspectives have fallen short of their potential to actively bridge conceptual constructs and, by extension, intervention approaches, across different levels of population impact. Theoretical and conceptual perspectives that explicitly span levels of impact offer arguably the greatest potential for achieving scientific insights that may in turn produce the largest population health effects. Examples of such “bridging” approaches include theories and models that span behavioral + micro-environment, behavioral + social/cultural, and social + physical environment constructs. Several recommendations are presented related to opportunities for leveraging theories to attain the greatest impact in the population health science field. These include applying the evidence obtained from person-level theories to inform methods for positively impacting the behaviors of community gatekeepers and decision-makers for greater population change and reach; leveraging the potential of residents as “citizen scientists”--a resource for enacting behavioral health changes at the individual, environmental, and policy levels; using empirical observations and theory in equal parts to build more robust, relevant, and solution-oriented behavior change programs; exploring moderators and mediators of change at levels of impact that go beyond the individual; and considering the circumstances in which applying conceptual methods that embrace a “complexity” as opposed to “causality” perspective may lead to more flexible and agile scientific approaches that could accelerate both population-relevant discoveries and applications in the field. The commentary closes with suggestions concerning additional areas to be considered to facilitate continued advances in the health behavior field more generally to attain the greatest impacts on population health.

## Background

Theory has long been used as a tool in the behavioral health field for organizing major putative constructs and their inter-relationships in explaining the potential drivers underlying key behaviors in the health promotion arena. The aim of this commentary is to highlight several directions in which the behavioral health field may profitably focus in expanding the potential impacts of theory-driven health promotion for influencing population health. Through seeking out theories and approaches that actively span levels of impact and accelerate more robust and translatable interventions, the field may continue to broaden its influences on population-wide health.

As the social, cultural, and environmental factors impacting health behaviors have grown in complexity, the contexts and constraints surrounding health behavior change have become increasingly multi-faceted. Theory can aid understanding both of how the world works and how potentially it can be improved, particularly if it embraces the multi-dimensional inputs that shape people’s lives. Yet, despite efforts to expand theoretical applications beyond primarily one impact level (e.g., individual level, environmental level), much of our theoretical work continues to have its epicenter anchored within the disciplinary “homes” in which we were academically raised. This, in turn, may narrow our ability to develop behavioral solutions that reach the larger population. For example, theories aimed at integrating different perspectives often remain embedded in primarily one impact level (e.g., the individual) [1, 2]. And while mobile technologies have brought more acuity to the contexts within which individuals’ health behaviors are embedded, such data often tend to maintain our focus at the personal level of influence, as observed in the Quantified Self movement [3]. Similarly, while ecological models have gained acceptance as multi-level alternatives to person-level approaches, much of the work from such models has strongly favored environmental influences. Less systematic focus has been aimed at person x environment (including policy) interactions, with less emphasis on applying theories that actively bridge perspectives across levels or facilitate multi-dimensional interventions (although this is beginning to change [4]). Examples of such person x environment interactions have been reported for neighborhood road networks where, for instance, cul-de-sacs have been associated with less physical activity among adults but more activity among youth [5].

To truly have a population health impact in the behavioral health field, theoretical approaches that embrace complexity and create multi-level solutions will likely be needed. What follows are some recommendations in this area.

### Further explore theories and models that explicitly span levels of influence

**Fig. 1:**
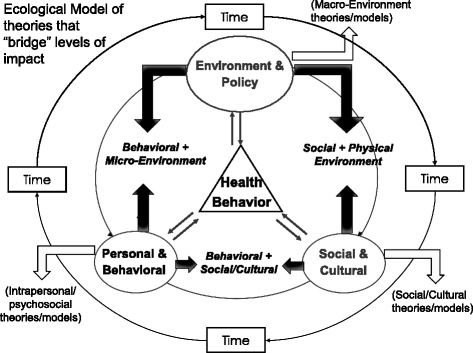
Ecological model of theories that "bridge" levels of impact

A next step in ecological model applications is to seek theories and perspectives that actively “bridge” multiple impact levels (Fig. 1). Investigators have embraced theories that connect behavioral with social/interpersonal constructs (e.g., social cognitive theory, social influence theory) and behavioral with micro-environmental constructs (operant conditioning, social cognitive theory, behavioral economics). However, there are additional theories and perspectives that can help to span broader macro-environmental levels of impact, such as behavioral plus social-cultural perspectives (e.g., individualism-collectivism; social identity; communication theories) and social plus physical environmental concepts (e.g., neighborhood disorder; “eyes on the street” environmental design paradigms to prevent crime) [6]. In addition to using constructs from such conceptual approaches as predictors of health behavior, actively pursuing the linkages among such multi-level constructs may stimulate more robust intervention development across diverse populations.

### Embrace the “reciprocal determinism” of theory and empirical observation in moving the field

Discourses on theory often neglect the foundational role that empirical observations play in developing and applying theory to solve real-world problems. To date, the dominant behavioral theories often do not effectively address time elements, cohort effects, or larger cultural or physical environmental contexts in ways that, informed by empirical observation, are sufficiently specific or actionable from a population health perspective. For example, while social cognitive theory emphasizes the reciprocal determinism between personal, behavioral, and environmental contexts, it has traditionally been microenvironments, as opposed to the larger-scale built and social environmental contexts, that have been studied.

Given the intricacies of understanding multi-level influences, it may be useful to further consider the role of “working schemas”, based on observations in the field, that may not rise to the stature of “theory” but may inspire pragmatic multi-level interventions [7].

### Apply thinking that embraces complexity

Behavioral health research often reflects a set of characteristics more appropriate to understanding relatively simple (e.g., psychosocial factors as pre-eminent) or complicated (e.g., importance of both the individual and environment) phenomena. Yet, health behaviors, by their nature, are more accurately described as “complex” [8]. Complex systems tend to be, among other things, heterogeneous in relation to targets and contexts of change; nonlinear with respect to variables that contribute to the behavior; unpredictable; dynamic; and having elements that are interdependent, with feedback loops [8]. For example, as cars become dominant in a locale, walkability often decreases, which in turn creates further need for motorized transport [8]. Some researchers have called for new models beyond traditional ecological frameworks that embrace complexity, such as “Foresight” mapping, which highlights the *connections* among multi-level factors that are part of a health behavior’s causal web [9]. Such models await empirical application and support.

### Seek intervention solutions that explicitly traverse levels of impact

**Fig. 2:**
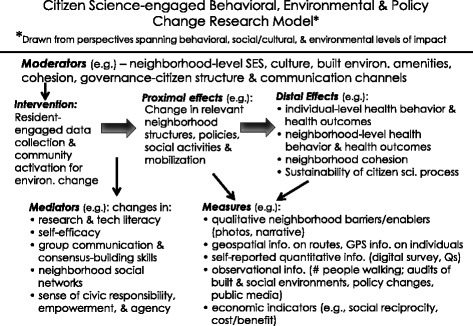
Citizen science-engaged behavioral , environmental, and policy change research model

Seeking ways in which individuals, and the theories generated in studying them, can become change agents for broader-level health behavior solutions deserves further investigation. An example of such an approach is the application of individual-level behavior change theories to the gatekeepers and decision-makers that materially influence, through their own behaviors, the local, regional, and national environments and policies that significantly impact behavioral health. Such applications can occur through the ways in which scientists communicate health challenges, research findings, and promising solutions to decision-makers and the public. Multi-level solution development can also occur through leveraging the potential of residents as “citizen scientists” in systematically assessing local barriers to and enablers of healthy lifestyles. Through such resident-engaged activities, mutually acceptable solutions can be developed with local stakeholders and researchers [10–12]. Such activities, informed by theoretical perspectives drawn from individual, social, and environmental research, have the potential to reach groups most vulnerable to health, social, and environmental inequities, as well being potentially scalable. Notably, instead of focusing on the health behaviors of the individual, individuals learn how to change aspects of their local physical and social environments that in turn can promote their own, arguably more sustainable, healthy lifestyles in addition to those of others sharing those environments. Figure 2 summarizes a general scientific framework for such citizen science models.

### Explore moderators and mediators of change at levels of impact beyond the individual

The field can benefit from further consideration of baseline moderators of intervention effects and mediators of change that, while commensurate with the theory being applied, go beyond traditional sociodemographic characteristics and psychosocial pathways. For instance, for theories emphasizing social and related environmental contexts (e.g., social influence theory, social cognitive theory), examples of higher-level variables that could potentially inform explorations of baseline moderators and/or mediators of change include larger-scale social environmental factors (e.g., cultural norms, shared perceptions of social stigma, neighborhood cohesion), as well as built environmental and regulatory factors [13]. The specific inclusion of such higher-order influences in moderator/mediator explorations may provide the types of multi-dimensional insights that could in turn inform more impactful and sustainable interventions.

### Facilitate continued conceptual advances in the field to impact population health

There are a number of other areas of potential relevance to theory in enabling broader population impact that deserve continued attention. While a fuller discussion of such areas is beyond this article’s scope, they include increasing our understanding of cultural factors in delineating the specificity versus generalizability of theories for populations worldwide; shaping theories to become sufficiently nimble and dynamic to better inform information technology interventions; taking full advantage of “big data” modeling and analytic techniques to further advance theory development for complex data sources, constructs, and populations; applying cutting-edge, theoretically-derived intervention design methods (e.g., iterative design, adaptive interventions, pragmatic trials) of particular relevance to real-world interventions; and applying theory to better inform the “whiches conundrum” (which interventions, for which people, under which circumstances) to achieve more enduring population-wide intervention success.

## Conclusions

In further exploring theoretical frameworks that explicitly drive the application of constructs and development of interventions that transcend levels of impact, we may better realize the full potential of behavioral health science in making substantive and durable contributions to population health.
